# A Novel Three-Dimensional Culture Device Favors a Myelinating Morphology of Neural Stem Cell-Derived Oligodendrocytes

**DOI:** 10.3389/fcell.2021.759982

**Published:** 2021-10-01

**Authors:** Alessandra Flagelli, Olivia Candini, Stella Frabetti, Massimo Dominici, Luciana Giardino, Laura Calzà, Vito Antonio Baldassarro

**Affiliations:** ^1^Interdepartmental Center for Industrial Research in Life Sciences and Technologies, University of Bologna, Bologna, Italy; ^2^Rigenerand Srl, Modena, Italy; ^3^Division of Oncology, Department of Medical and Surgical Sciences for Children and Adults, University-Hospital of Modena and Reggio Emilia, Modena, Italy; ^4^Department of Veterinary Medical Science, University of Bologna, Bologna, Italy; ^5^IRET Foundation, Bologna, Italy; ^6^Department of Pharmacy and BioTechnology, University of Bologna, Bologna, Italy; ^7^Montecatone Rehabilitation Institute, Imola, Italy

**Keywords:** oligodendrocyte, oligodendrocyte precursor cell (OPC), culture systems, three-dimensional culture (3D culture), *in vitro* myelination

## Abstract

The complexity of the central nervous system (CNS) requires researchers to consider all the variables linked to the interaction between the different cell inhabitants. On this basis, any *in vitro* study of the physiological and pathological processes regarding the CNS should consider the balance between the standardization of the assay and the complexity of the cellular system which mimics the *in vivo* microenvironment. One of the main structural and functional components of the CNS is the oligodendrocyte precursor cell (OPC), responsible for developmental myelination and myelin turnover and repair during adulthood following differentiation into mature oligodendrocytes. In the present brief research report, we describe a 3D culture tool (VITVO) based on an inert and biocompatible synthetic polymer material scaffold, functionalized with laminin coating, and tested as a new culture microenvironment for neural stem/precursor cell (NSPC) differentiation compared to standard 2D cultures. NSPCs spontaneously differentiate in the three neural lineages (neurons, astrocytes and OPCs), identified by specific markers, along the fibers in the 3D structure. Analysis of the mRNA levels for lineage differentiation markers reveals a higher expression compared to those seeded on a 2D surface, suggesting an acceleration of the differentiation process. We then focused on the oligodendroglial lineage, showing that in VITVO, mature oligodendrocytes exhibit a myelinating morphology, proven by 3D image elaboration, linked to a higher expression of mature oligodendrocyte markers. This preliminary study on an innovative 3D culture system is the first robust step in producing new microenvironment-based strategies to investigate *in vitro* OPC and oligodendrocyte biology.

## Introduction

*In vitro* modeling of the central nervous system (CNS) is still a significant challenge, due to its complex cellular composition, cell-specific microenvironments, extracellular matrix composition, cell-to-cell contacts and a myriad of soluble signals, all cues which are organized in a tightly regulated three-dimensional (3D) structure (Nikolakopoulou et al., 2021).

The challenge of modeling dynamic events in the CNS, such as myelination and remyelination by oligodendrocytes (OLs), is even greater. OLs are one of the largest CNS cell populations, and derive from multipotent neural stem cells, which differentiate through the oligodendrocyte precursor cell (OPC) to premyelinating and mature myelinating OLs. During fetal and early postnatal life, OPCs are in charge of developmental myelination and generation of the pool of precursors which colonizes the entire white and gray matter of the adult CNS. This pool is responsible for myelin turnover and repair in adulthood, ensuring proper morphology and function of the white matter (WM) (van Tilborg et al., 2018; Kuhn et al., 2019). In addition to its physiological role, WM dysfunction is the key pathogenic event in demyelinating diseases, and its major role in CNS injuries and neurodegenerative diseases is also beginning to emerge. Due to the capability of OPCs to repair myelin damage and restore its function, these cells are also regarded as a potential target for the development of therapeutic strategies (Bae et al., 2020).

Bidimensional (2D) cultures, the most widely used *on dish* system, are easy to handle and highly reproducible, but do not gain relevant translational power, contributing to the high failure rate and a time to approval in drug development for CNS targets which is 38% longer than for other applications (Bang et al., 2019; Maoz, 2021). Researchers should therefore consider the equilibrium between the reproduction of microenvironment complexity and the reliability of a simplified system when seeking to develop reliable *in vitro* CNS models.

Creating such a model of OPC differentiation and myelination is a key challenge in the construction of a pipeline for the study of the biology of this recently discovered cell, one able to model pathological conditions and aid in drug discovery (Baldassarro et al., 2021). In this study, we investigated OPC differentiation by comparing standard culture conditions and a laminin-functionalized 3D culture tool based on PBT (polybutylene terephthalate) fibers (VITVO^®^). OPCs were derived from fetal neural stem/precursor cells (NSPCs) using a standardized method which allows generation of the three neural lineages (neurons, astrocytes, and oligodendrocytes). We focused on OPC maturation, based on qualitative cell morphology observation and mRNA quantitative analysis for differentiation and myelin protein encoding genes, and we describe that differentiating OLs in VITVO show a myelinating-like morphology and a higher expression level of OL lineage and maturation-related genes (*Olig1*, *Olig2*, *klf9*, *Mbp*) compared to conventional 2D cultures.

## Methods

### Neural Stem Cell Isolation and Culture

**FIGURE 1:**
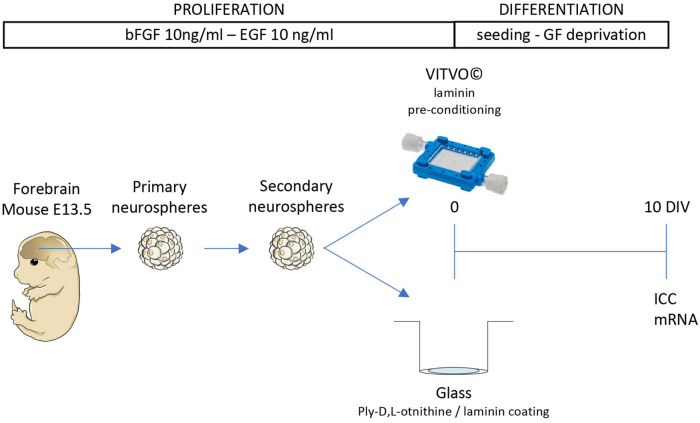
Cell culture procedure. Schematic representation of the experimental protocol. Cells were isolated from the forebrain of the fetal mouse (E13.5) and maintained as 3D suspension cultures (neurospheres). After the formation of the secondary neurospheres, cells were seeded in VITVO pre-conditioned with laminin or glass coated with poly-L-ornithine and laminin. After 10 days in culture (DIV), cells were stained for immunocytochemistry (ICC) or gene expression analysis.

Fetal NSPCs were isolated following previously published protocols (Baldassarro, 2021) with a number of modifications (Figure 1).

All embryos were collected from pregnant female mice at 13.5 days postcoitus and placed in a 50 ml tube containing PBS and penicillin/streptomycin (P/S, Cat. 15140148, Thermo Fisher Scientific, Waltham, MA, United States) 100 U/100 μg. Under a dissection microscope, the brains were removed using a lancet and placed upright on the plate. The meninges were carefully detached using forceps, the olfactory bulbs removed, and the forebrains collected in a 1.5 ml tube.

The PBS was removed and the tissues incubated with the non-enzymatic dissociation buffer (Cat. 13150016, Sigma-Aldrich, St. Louis, MO, United States). After 15 min incubation at 37°C, the tissues were pipetted several times for mechanical dissociation. After 5 min of centrifugation at 400 × g, the cellular pellet was resuspended in serum-free NSPC medium (DMEM/F12 GlutaMAX 1x, Cat. 10565018; 8 mmol/L HEPES, Cat. 15630-056; 100 U/100 μg P/S; 1 × B27, Cat. 12587010; 1 × N-2, Cat. 17502048; 10 ng/ml bFGF, Cat. PHG0024; 10 ng/ml EGF, Cat. PHG6045; Thermo Fisher Scientific), and the cells plated at a density of 10 cells/μl in a T25 flask (Corning, New York, NY, United States) following cell count. The medium was changed every 3 days.

To obtain the secondary neurospheres, the cells were centrifugated at 400 × g for 5 min and resuspended in 1 ml of medium, then counted and re-plated at the same density and under the same conditions.

Before seeding, the VITVO device (Rigenerand srl, Medolla, MO, Italy) and glass coverslips were prepared using specific coatings. In particular, the glass coverslips were pre-treated with poly-L- ornithine (50 μg/ml; Cat. P0421, Sigma-Aldrich) overnight at room temperature and washed three times in sterile distilled water. Both the coverslips and the VITVO devices were then coated with 1.5 ml of 5 μg/ml laminin (Cat. L2020, Sigma-Aldrich) and incubated in a humidified incubator at 37°C with 5% of CO_2_ for 3 h.

### VITVO Device

The VITVO device is formed by a continuous perimeter frame in TPE (thermoplastic elastomer) equipped with two transparent oxygenation optical membranes that allow gas exchange and visibility. The 3D inner core is a fiber-based matrix in PBT, an inert and biocompatible synthetic polyester, with a thickness of 400 μm. The fibers have a diameter of 1,7 μm, and they are randomly distributed with an inter-distance among fibers of 10 μm (median value).

Internally, VITVO consists of two semi-chambers separated only by the 3D matrix. Each semi-chamber has a port that acts as both an inlet and an outlet, depending on the fluid flow. Cells colonize the matrix laying on fibers, adhering on them or forming aggregates in the empty volume between fibers which is 90% of the total volume.

The cell suspension, the medium used for the culture and the laminin conditioning liquid was injected with a syringe connected to one of the two ports. Once injected, the liquid enters the first semi-chamber and then passes through the second one throughout the 3D matrix which acts as a filter, retaining cells.

Any air bubble was removed from with a syringe, putting in vertical the device allowing the air reaching one of the two ports.

### Immunocytochemistry

Indirect immunofluorescence procedures were used to identify neural precursors (nestin), neurons (B-III-tubulin), astrocytes (GFAP) and mature oligodendrocytes (CNPase, MBP).

Cells were washed in PBS and fixed in 4% paraformaldehyde in 0.1 M Sørensen phosphate buffer for 20 min at room temperature. Cells were then blocked with 1% Donkey Normal Serum (Cat. 1164101, Sigma-Aldrich) in 0.3% PBS/Triton-X 100 (Cat. 9002-93-1, Merck, Darmstadt, Germany) for 1 h at room temperature, then incubated overnight at 4°C in a humid atmosphere with the primary antibody diluted in 0.3% PBS/Triton-100.

For the cells cultured in the VITVO, the fixation and washing steps were performed directly inside the device. The outer optical membrane was then cut with a lancet and the PBT scaffold extracted, cut into pieces and placed in a well of a 24 multiwell culture plate. From this step onward, both the PBT scaffolds and glass coverslips were processed in the same way.

The following primary antibodies were used: mouse anti-nestin (1:200, Cat. LV1797294 Millipore, Burlington, MA, United States), mouse anti-β-III-tubulin (1:500, Cat. SC58888, Santa Cruz Biotech, Dallas, TX, United States), rabbit anti-GFAP (1:1,000, Cat. Z0334, Dako), mouse anti-CNPase (1:300, Cat. MAB326R Millipore) and rabbit anti-MBP (1:300, Cat. A0623, Agilent Technologies Dako, Santa Clara, CA, United States). After rinsing in PBS (2 × 10 min), cells were incubated with fluorochrome-conjugated secondary antibodies, and diluted in 0.3% PBS/Triton-X 100 for 30 min at 37°C. The following secondary antibodies were used: Cy2-conjugated donkey anti-mouse (1:500, Cat. AB2340827, Jackson laboratories, Bar Harbor, ME, United States) and RRX-conjugated donkey anti-rabbit (1:500, Cat. 711295152, Jackson laboratories).

For nuclear staining, 1 μg/ml Hoechst 33258 (Cat. H1398, Thermo Fisher Scientific), was added during incubation of the secondary antibodies. After rinsing in PBS, both the coverslips and the PBT scaffold were mounted on microscopy slides in 0.1% glycerol/1,4-phenilendiamine (Cat. 56815, Cat. 106503, Sigma-Aldrich).

### Confocal Microscopy and 3D Image Analysis

Confocal microscopy was used to study the neural stem cell-derived lineages and cell morphology. Slides were analyzed with a Nikon Ti-E fluorescence microscope, connected to an A1R confocal system (Nikon, Minato, Tokyo, Japan) consisting of a series of diode lasers with an output wavelength of 405 nm, an air-cooled argon-ion laser system with 488 nm output, and a yellow diode-pump solid-state laser system with a 561 nm wavelength output. Images were acquired using a 40x lens with 1024 × 1024 resolution, and all z-stacks were collected in compliance with optical section separation (z-Interval) values suggested by the NIS-Elements AR 3.2 software (1 μm).

### Gene Expression Analysis

For gene expression studies using qPCR, total RNA isolation was extracted using the RNeasy Micro Kit (Cat. 74004, Qiagen, Hilden, Germany), following the manufacturer’s instructions. RNA was eluted in RNase free water (14 μl) and concentrations estimated through absorbance values at 260, 280, and 320 nm (NanoDrop 2,000 spectrophotometer, Thermo Fisher Scientific).

First strand cDNAs were obtained using the iScript^TM^ gDNA Clear cDNA Synthesis Kit (Cat. 1725025BUN, Bio-Rad, Hercules, CA, United States), and an RNA sample with no reverse transcriptase enzyme in the reaction mix was processed as a no reverse transcription control.

Semi-quantitative real-time PCR reactions were performed in a final volume of 20 μl consisting of 1 × SYBR Green qPCR master mix (Cat. 1725274, Bio-Rad) and 0.5 μM forward and reverse primers, using the CFX96 real-time PCR system (Bio-Rad). The no-reverse transcriptase sample was processed in parallel with the others and tested by real-time PCR for each pair of primers used to prevent any possible contamination. All primers used were designed using Primer Blast software and synthesized by IDT (Coralville, IA, United States), and *Gapdh* was used as housekeeping gene to normalize the amount of reverse- transcribed RNA used for PCR. The following primers were designed and used for the reactions: *Gapdh* (FW: GGC AAG TTC AAT GGC ACA GTC AAG; REV: CAT ACT CAG CAC CAG CAT CAC); *Gfap* (FW: AGT GGT ATC GGT CCA AGT TTG C; REV: TGG CGG CGA TAG TCA TTA GC); *Klf9* (FW: AGT GGC TTC GAA GGG GAA AC; REV: TCC GAG CGC GAG AAC TTT TT); *Mbp* (FW: GCC TGT CCC TCA GCA GAT TT; REV: GTC GTA GGC CCC CTT GAA TC); *Ntf3* (FW: TGC CCA AAG CAG AGG CAC CC; REV: GCA GGA CCC GGG GCG AAT TG); *Olig1* (FW: CCG CCC CAG ATG TAC TAT GC; REV: AAC CCA GCT CAT ACA GC); *Olig2* (FW: TGG CCC CAG GGA TGA TCT AA; REV: TTA CAG ACC GAG CCA ACA CC).

All PCR reactions were performed according to the following thermal profile: a first step of denaturing (95°C, 2 min) and 40 cycles of amplification (95°C for 15 s and 60°C for 60 s). The melting curve of amplified products followed this temperature/time scheme: heating from 55 to 95°C with a temperature increase of 0.5°C/sec.

Primer efficiency values for all primers were 95–102%, thus the 2^(–ΔΔ^ ^CT)^ method was used for the calculation of gene expression.

### Statistical Analysis

Data is reported as mean ± SEM. Prism software (v.8; GraphPad Software, San Diego, CA, United States) was used for statistical analyses and graph generation. Data was collected from at least three independent experiments, Student’s *t*-test was used for the analysis, and the results were considered significant when the probability of their occurrence as a result of chance alone was < 5% (*p* < 0.05).

## Results

### Neurospheres Integrated Into the 3D Device

**FIGURE 2:**
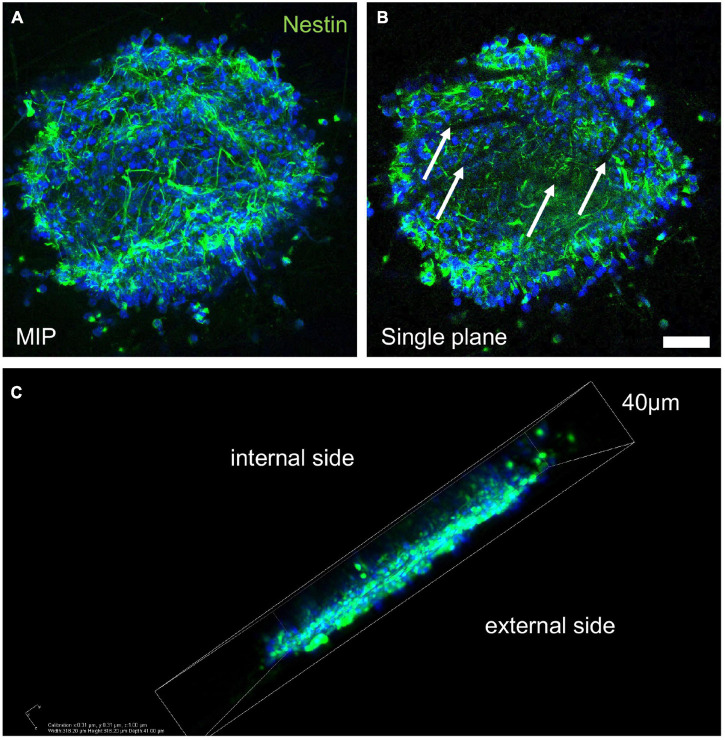
Sphere integration into the three-dimensional PBT scaffold. **(A–C)** Representative images of confocal z-stack derived maximum intensity projection (MIP; **A**), single plane picture **(B)**, and 3D reconstruction **(C)** of a secondary neurosphere seeded in VITVO and stained by immunocytochemistry for nestin marker, after 3 days in culture. White arrows in B indicate the PBT fibers. Scale bar **(A,B)** 30 μm.

We first investigated the biocompatibility of the neurospheres using VITVO by injecting secondary spheres into the 3D device. Three days after seeding, the spheres integrated into the fiber net of the device. At this stage, the spheres consisted mainly of nestin-positive neural precursors (Figure 2A) and surrounded the fibers, as shown by the confocal imaging of a single focal plane, where the fibers appeared as dark filamentous spaces within the sphere (Figure 2B, white arrows). However, the spheres deposited mainly on the surface of the PBT mesh, with only few aggregates moving inside the scaffold (Figure 2C).

### The 3D Device Accelerated Neuron and Astrocyte Maturation of Neural Stem/Precursor Cell-Derived Cells

**FIGURE 3:**
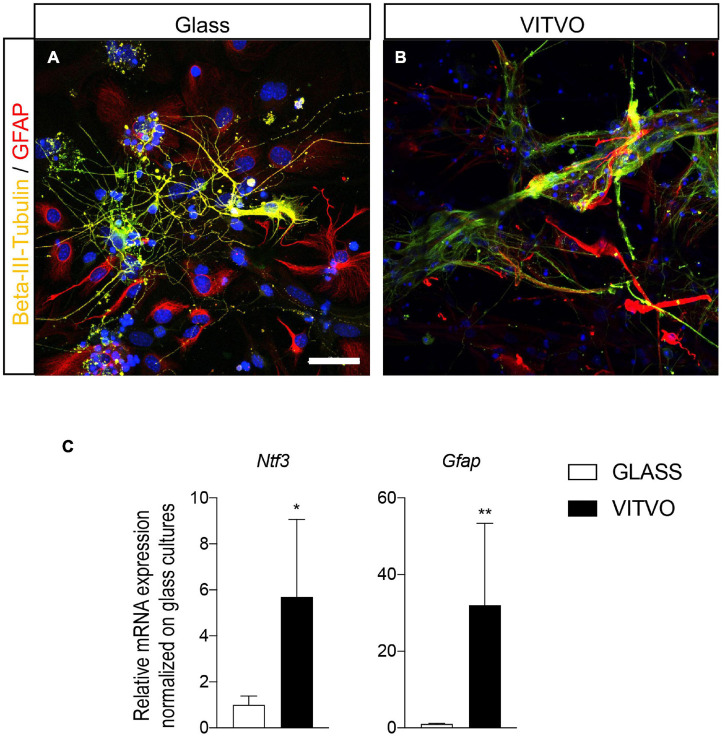
**(A,B)** Representative images of neurons and astrocytes seeded on laminin coated glass **(A)** or in VITVO device **(B)** after 10 days in culture. Scale bar: 50 μm. **(C)** Graphs represent expression of *Ntf3* and *Gfap* genes in neural stem cells seeded on glass or laminin after 10 days in culture. Statistical analysis: Student’s *t*-test. Asterisks represent differences between cultures seeded on glass or in VITVO (*p < 0.05; **p < 0.01).

After 10 days in culture on glass coverslips or in the VITVO device, lineage-specific markers were used to characterize differentiation from the NSPC culture. Neurons, identified by β-III-tubulin, and astrocytes, identified by GFAP, showed a similar cell body and elongation morphology when conventionally cultured (Figure 3A) and cultured in the 3D device (Figure 3B), while in the latter, the cell body tended to lie between or on the fibers, and respective elongations, including neurites, extended over the scaffold fibers.

To establish whether these morphological aspects matched with a gene expression switch supporting lineage specification and differentiation, we analyzed the mRNA expression level of genes fundamental for lineage-specific differentiation and terminal differentiation. We analyzed *Ntf3*, a critical factor which participates in neuronal differentiation, and the astrocyte marker *Gfap* (Figure 3C). Cultures seeded in the VITVO showed a higher expression of both *Ntf3* (Student’s *t*-test, *p* = 0.0374) and *Gfap* (*p* = 0.0044) genes after 10 days of spontaneous differentiation compared to 2D coverslip cultures. To be noted is that, even reaching the statistically significance, the gene expression analysis showed high variability between VITVO replicates.

### The 3D Device Favored a Promyelinating Phenotype of Neural Stem/Precursor Cell-Derived Oligodendrocytes

**FIGURE 4:**
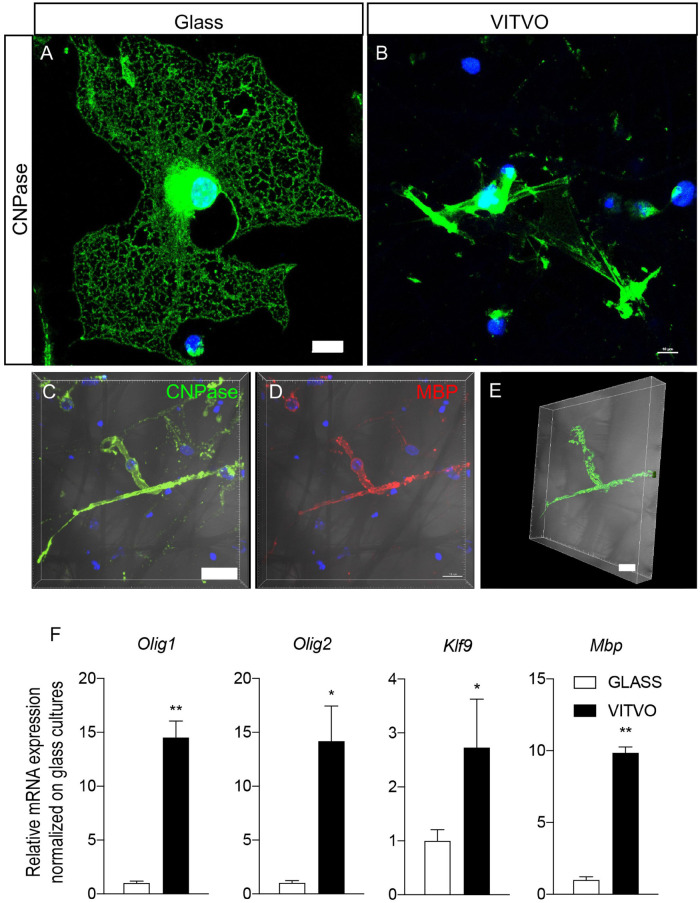
**(A,B)** Representative images of mature oligodendrocytes seeded on laminin coated glass **(A)** or in VITVO **(B)** after 10 days in culture. Scale bar: 10 μm. **(C–E)** Representative confocal images of neural stem cell-derived mature oligodendrocyte stained with CNPase **(C)** or MBP **(D)** showing the maximum intensity projection of the z-stack, and the IMARIS software elaboration **(E)**. The bright field is included in the pictures. Scale bar: 15 μm **(C,D)**; 10 μm **(E)**. **(F)** Graphs represent expression of *Mbp*, *Klf9*, *Olig1*, and *Olig2* genes in neural stem cells seeded on glass or laminin after 10 days in culture. Statistical analysis: Student’s *t*-test. Asterisks represent differences between cultures seeded on glass or in VITVO (*p < 0.05; **p < 0.01).

Mature OLs, as identified by CNPase immunoreactivity, showed significant differences in cell morphology in the two culture conditions (Figures 4A,B). On the 2D surface, OLs developed the typical spider net-like membrane generated by a wide cell membrane expansion all around the cell body (Figure 4A), in contrast to the 3D culture system, where mature oligodendrocyte cell bodies were smaller, with extensions touching different fibers and resembling a myelin-like structure (Figure 4B). To further investigate this aspect, we used confocal imaging followed by IMARIS software 3D isosurface reconstruction of cells immunoreactive for mature oligodendrocyte markers (CNPase, Figure 4A, MBP, Figure 4B) with the cell body protrusions wrapping the fiber of the scaffold (Figures 4C,D), as also shown by isosurface reconstruction (Figure 4E).

We then analyzed the gene expression of specific markers for oligodendrocyte lineage and terminal differentiation, particularly *Olig1* and *Olig2*, transcription factors which induce oligodendroglial lineage; *Klf9, a* transcription factor which regulates the transactivation of thyroid hormone-responsive promoters of genes responsible for oligodendrocyte terminal differentiation, and *Mbp*, which encodes for myelin basic protein, the most abundant protein in mature myelin. We demonstrated that cells differentiating on VITVO devices showed a higher expression of all these genes (Figure 4F) as follows (Student’s *t*-test): *Mbp*, *p* = 0.0031; *Klf9*, *p* = 0.049; *Olig1*, *p* = 0.0057; *Olig2*, *p* = 0.0115.

## Discussion

*In vitro* models of NSPC differentiation and maturation poorly reflect *in vivo* biology, particularly as regards OLs. This is mainly due to the complex lineage progression of these cells, required to move in a dish from precursors to mature cells, as defined by morphology, molecular signature, and attitude to envelop axonal fibers. Conventional 2D cultures of NSPC-derived cells or co-culture-based models poorly mimic the *in vivo* microenvironment which drives the entire differentiation and maturation process, and many 3D-based models have been proposed over the last few years to overcome these limitations. While many of these systems are useful for exploring the molecular differentiation process, most of them fail to mimic the myelination process, e.g., wrapping of the axon by the OL cytoplasm.

The aim of the present study was to explore the possibility of differentiating NSPC-derived oligodendrocytes in a simple 3D microenvironment to overcome the differentiation and morphological bias of the conventional 2D cultures.

Here we showed that culturing NSPC-derived cells in a 3D device based on PBT fibers accelerated differentiation compared to conventional 2D cultures along the three expected lineages, i.e., neurons, astrocytes and oligodendrocytes, as established by morphology and gene expression signature. Moreover, the mechanical cue provided by the fiber mesh which mimics axon fibers (fiber diameters: 0.2–2 μm) strongly accelerated the maturation of the OPCs, which differentiated into OLs which spontaneously wrapped the fibers in a myelination-like morphology, suggesting that this device can be further developed and standardized as an *in vitro* system for axon myelination assays. Notably, the option of connecting the device to a microfluidic system could further reduce the NSPC *in vitro* differentiation time, as demonstrated in 3D cultures of other cell types (Lovecchio et al., 2020). This small, portable bioreactor has been proposed as an efficient alternative to standard 2D cultures as preclinical platform for drug testing, gene therapy and immune-oncology studies (Candini et al., 2019; Corallo et al., 2020).

To increase the microenvironmental compliance of the NSPCs and derived cells, we functionalized the plastic-based mesh with a laminin coating, and used the conventional culture medium. Laminin matrices enhanced NSPC migration, expansion, differentiation into neurons and astrocytes, and elongation of neurites from NSPC-derived neurons (Liu et al., 2020), an effect possibly mediated by the α6 integrin subunit (Flanagan et al., 2006), thus confirming the importance of cell-matrix interaction in a microenvironment mimicking the NSPC niche. In addition, laminins expressed around blood vessels positively regulated the migration and survival of OPCs through the integrin β1-FAK pathway (Suzuki et al., 2019). Moreover, laminin is the most used coating for neurosphere-derived and primary OPCs (Chen et al., 2007; Lourenço et al., 2016), as the main extracellular matrix mediator of their adhesion and differentiation (Liu et al., 2020). Since the aim of the present study was to compare the 2D conventional cultures with the differentiation in VITVO, we did not focus on different coatings in the 3D microenvironment, but we used the same conditions for both cultures, reducing the numbers of possible variables.

Both spheroids and single cells interacted with the PBT fibers and integrated into the three-dimensional space of the device, in which all three lineages underwent accelerated maturation compared to conventional 2D cultures, as confirmed by the morphological features and respective molecular signatures. Astrocyte and neuron morphology was similar in 2D and VITVO-assisted 3D cultures, but gene expression suggested an accelerated differentiation, given that expression of *Ntf3*, a key regulator of neuronal differentiation (Lin et al., 2018), and *Gfap*, indicating astrocyte differentiation (Ito et al., 2016), was more than 6 and 30 times higher, respectively, in VITVO compared to conventional cultures.

As a recent example of the effect of the 3D microenvironment, 3D cultures of human NSPCs on hydrogels showed improvements in proliferation and differentiation, directed more toward neurons and oligodendrocytes, compared to 2D cultures (Seidlits et al., 2019). Moreover, 3D models have also been used to investigate the role of ligand presentation in promoting oligodendroglial lineage specification (Wen et al., 2019), and for large-scale production of OPCs for the development of CNS disease treatments (Rodrigues et al., 2011).

Conventional 3D systems not assisted by axons or axon-like structures, however, give an incomplete picture, indeed the most significant difference between conventional and VITVO cultures was observed in oligodendrocyte maturation. Not only were the OL lineage induction genes (*Olig1* and *Olig2*; Meijer et al., 2012), OPC differentiation genes (*Klf9*; Dugas et al., 2012) and OL maturation genes (*MBP*; Simankova et al., 2021) strongly up-regulated in the 3D compared to the 2D culture (around 15, 15, 3, and 10 times, respectively), but the morphology of NSPC-generated oligodendrocytes was completely different in conventional and 3D cultures. While the OL plasma membrane showed the typical spider web-like membrane around the cell body in a conventional 2D culture, in the 3D device the OL cell bodies lay between fibers of the PBT net, and the plasma membrane protrusions wrapped individual fibers of axon-compatible diameter, reflecting a myelinating-like morphology (Barateiro and Fernandes, 2014).

The importance of topographical and mechanical cues to establish the myelination potential of differentiated OLs is a recognized need for *in vitro* myelination assays, since the molecular signature *per se* is insufficient to prove that the OL recognizes the target and is able to wrap it by multiple concentric layers of compact membrane. Neuron-OL co-cultures are complex and not easily standardizable, while nanomaterial-assisted 3D cultures provide a useful and easy-to-use tool (Lee et al., 2013).

## Conclusion

In conclusion, a 3D microenvironment is essential for the proper differentiation and maturation of neural cells, shifting research toward the development of systems with different levels of complexity (Baldassarro et al., 2016) to avoid the production of immense amounts of discrepant data with low translational power, typical of the 2D-based gold standards for pre-clinical neurobiological research (Baldassarro et al., 2021). The main goal is to develop “microphysiological systems” to bridge the gap between these standardized 2D screening platforms and 3D systems which incorporate aspects of the tissue microenvironment (Anderson et al., 2021). In this preliminary study we described the first step of a simple and innovative 3D system for producing microenvironment-based strategies for OL studies, taking us another step closer to an ideal, yet still elusive *in vitro* model, one which strikes an optimal balance between a simplification of the model itself and the complexity of the myelination process.

## Data Availability Statement

The raw data supporting the conclusions of this article will be made available by the authors, without undue reservation.

## Author Contributions

OC, SF, MD, LG, and LC contributed to conception and design of the study. AF and VB performed the experiments, performed the statistical analysis, and wrote the first draft of the manuscript. OC, LC, and VB wrote sections of the manuscript. All authors contributed to manuscript revision, read, and approved the submitted version.

## Conflict of Interest

OC, SF, and MD were affiliated to Rigenerand Srl, the Company producer of the VITVO device. The remaining authors declare that the research was conducted in the absence of any commercial or financial relationships that could be construed as a potential conflict of interest.

## Publisher’s Note

All claims expressed in this article are solely those of the authors and do not necessarily represent those of their affiliated organizations, or those of the publisher, the editors and the reviewers. Any product that may be evaluated in this article, or claim that may be made by its manufacturer, is not guaranteed or endorsed by the publisher.
